# Evidence for benefits and risks of tadalafil as a non-prescription medicine: review and evaluation using the Group Delphi technique to achieve consensus amongst clinical experts

**DOI:** 10.3389/fphar.2023.1254706

**Published:** 2023-10-09

**Authors:** Kurt Miller, Uwe May, Wolf-Dietrich Beecken, Georgios Hatzichristodoulou, Michael Böhm, Stefan Fink

**Affiliations:** ^1^ Department of Urology, Charité Campus Benjamin Franklin, Berlin, Germany; ^2^ Department of Health Economics and Pharmacoeconomics, Fresenius University of Applied Sciences, Wiesbaden, Germany; ^3^ Department of Urology, Goethe University, Frankfurt, Germany; ^4^ Martha-Maria Hospital Nuremberg, Nuremberg, Germany; ^5^ Department of Internal Medicine, University of the Saarland, Homburg Saar, Germany; ^6^ State Pharmacists’ Association of Thuringia, Erfurt, Germany

**Keywords:** erectile dysfunction, non-prescription, phosphodiesterase type 5 inhibitor (PDE5 inhibitor), reclassification, tadalafil

## Abstract

An evidence-based consensus meeting was held with urologists, a pharmacist and a cardiologist to perform a structured benefit-risk analysis of reclassifying tadalafil, a phosphodiesterase type 5 (PDE5) inhibitor for treatment of erectile dysfunction (ED), to be available without prescription in Germany. As per the Brass process endorsed by regulatory authorities, an evidence-based Brass value tree was developed, which identified the incremental benefits and risks that should be considered above the safety and efficacy evidence required for prescription medicines. During the Group Delphi consensus meeting, the expert panel rated the likelihood and clinical impact of each benefit and risk on a scale of 0 (none) to 3 (high). Overall attribute scores were calculated from the product of the mean likelihood and mean clinical impact scores giving a possible score of 0–9. The overall benefit attribute scores ranged from 2.8 to 5.4. The overall risk attribute scores ranged from 0.2 to 2.2 though most were 1.0 or less (3 or more is generally considered to be of concern). On balance, the independent meeting scored the benefits of reclassification of tadalafil higher than the risks and considered the risk mitigation strategies of the packaging label and patient information leaflet (PIL) sufficient.

## 1 Introduction

There is a high prevalence of erectile dysfunction (ED) in Europe, with 42% (53.9 million) of men self-reporting difficulty achieving or maintaining an erection in the previous 5 months (Goldstein et al., 2020). In Germany, the figure is between 33% and 45%, which includes 5% of younger men aged 18 to 39 (Jannini et al., 2014; Briken et al., 2020; Goldstein et al., 2020). Despite the high prevalence, up to 81% of men do not seek treatment, so ED remains under-recognised, underdiagnosed and undertreated (Shabsigh et al., 2004; de Boer et al., 2005; Moreira et al., 2005; Nicolosi and et a l., 2006; Frederick et al., 2014; Jannini et al., 2014).

The phosphodiesterase type 5 (PDE5) inhibitor, tadalafil, is currently only available in Germany via a prescription. The aim of this study was to convene an expert panel to quantify the likelihood of occurrence and the potential clinical impact of each incremental benefit and risk identified for reclassification of tadalafil as a non-prescription medicine.

To ensure a systematic, transparent methodology was followed, a Brass analysis was undertaken (Brass et al., 2013). This structured process has been developed to help in complex decision-making for reclassification of prescription medications and is endorsed by regulatory authorities such as the UK Medicines and Healthcare products Regulatory Agency (MHRA) and the US Federal agency, the Food and Drug Administration (FDA) (Brass et al., 2011; Medicines and Healthcare products Regulatory Agency, 2021).

## 2 Materials and methods

**FIGURE 1:**
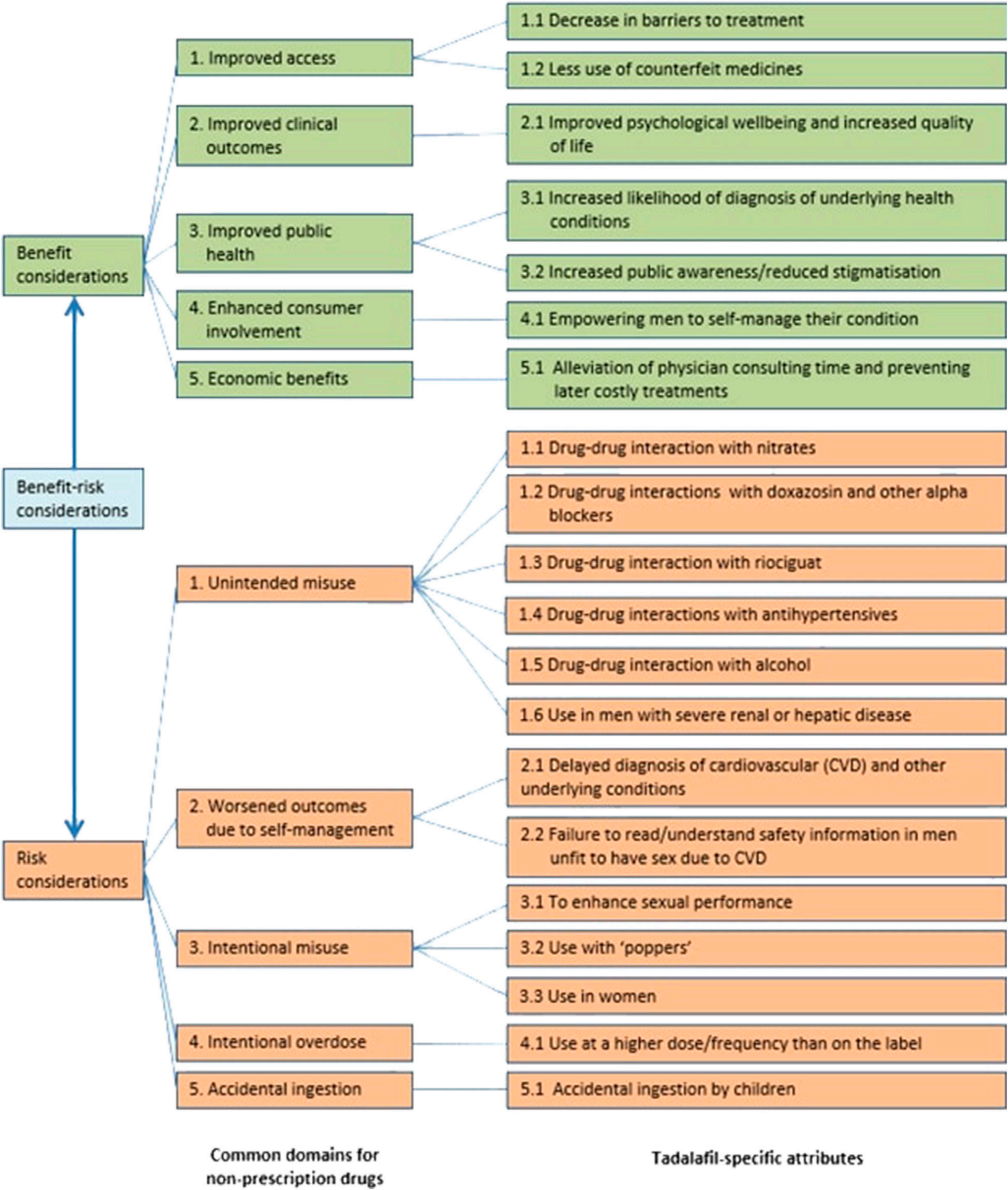
Brass value tree of incremental benefits and risks associated with non-prescription tadalafil.

The Brass analysis began with the development of a Brass value tree, which was informed by a comprehensive evidence review with clinical expert input (Figure 1) (Brass et al., 2011). The Brass value tree is a framework for identifying incremental benefits and risks that might arise in addition to the established efficacy and safety of the product as a prescription medicine according to the European Medicines Agency Article 71 (1) of Directive 2001/83/EC (EMA, 2006). Within each pre-defined major benefit and risk domain that should be considered for any non-prescription drug, specific attributes were identified for tadalafil. The Brass value tree and evidence for each incremental benefit and risk were circulated to an expert clinical panel prior to the consensus meeting.

The expert panel comprised four urologists, one of whom chaired the meeting, a cardiologist, a pharmacist, and the speaker was a clinical pharmacologist. This mix of specialties ensured that different important perspectives could be captured with the necessary clinical expertise to critically review the evidence, identify any additional benefits and risks, and understand the clinical impact of making a PDE5 inhibitor for the treatment of ED available without prescription. The objective of the meeting was to achieve an expert consensus on the benefits and risks; hence a Group Delphi technique was utilised. The speaker presented the evidence, it was discussed, and then the panel gave independent ratings on the likelihood and clinical impact of each incremental benefit and risk (Okoli and Pawlowski, 2004; Brass et al., 2013). Both the likelihood and clinical impact were scored from 0 (none) to 3 (high) or a “do not know” option. Results were fed back to the panel. Where consensus was not achieved, defined as scores more than 1 point apart, further open discussion was initiated. The rating was then repeated. Overall scores were calculated from multiplying the mean likelihood of behaviour or event and the mean clinical impact providing an overall benefit or risk score between 0 and 9. A Public Health consultant provided expertise in relation to the health economic and public health benefits but not for the clinical benefits and risks. The speaker and Public Health consultant participated in discussions but did not rate the risks and benefits. The meeting was thus chaired and run by the independent group of experts with no input or participation from pharmaceutical companies.

### 2.1 Evidence review

The evidence for each incremental benefit and risk domain identified in the literature review for tadalafil is summarised below in Tables 1, 2. Post-marketing safety data are reported using periodic safety update reports (PSUR) and periodic benefit-risk evaluation reports (PBRER) for tadalafil that have been submitted to regulatory authorities by Lilly as required by regulations since the first marketing authorisation for tadalafil on 15 October 2002 up to 15 October 2020.

**TABLE 1:** Evidence review summary of incremental benefit considerations.

Benefit consideration	Concern	Evidence
1 Improved access		
1.1 Decrease in barriers to treatment	• In Europe, 47%–81% of men with erectile dysfunction (ED) do not seek medical help Haro et al. (2006); Nicolosi and et al. (2006); European Association of Urology (2020)	• Access in a pharmacy overcomes the difficulty of obtaining a doctor appointment Pike (2019)
	• Only 7% of men are asked about sexual health by healthcare professionals (HCP) Gott et al. (2004); Moreira et al. (2005)	• Up to 50% of initial ED treatment discussions now occur in pharmacies in Spain and the United Kingdom Morales et al. (2010); Lee et al. (2021)
1.2 Less use of counterfeit medicines	• 30%–50% of men taking phosphodiesterase type 5 (PDE5) inhibitors are exposed to illicit preparations, which are unregulated products with inadequate or no labelling warnings Jack (2007); Jackson et al. (2010); Schnetzler et al. (2010); Campbell et al. (2012); United States Food and Drug Administration FDA (2015); The Local (2019)	• In the United Kingdom, the United Kingdom Medicines and Healthcare products Regulatory Agency (MHRA) considered a reduction in risks associated with counterfeit supplies of sildenafil to be a key benefit in making it available non-prescription Medicines and Healthcare products Regulatory Agency (2017a)
	• Concentrations of active ingredients in counterfeit pills range from 0% to 200% of the indicated strength Jackson et al. (2010); Campbell et al. (2012); Chiang et al. (2017)	• In New Zealand there was a reduction in counterfeit sildenafil packages intercepted at the border following reclassification to non-prescription status in 2014 The Pharmaceutical Journal (2018)
	• Toxic substances or adulterants include antidepressants, antibiotics, commercial-grade paints and printer ink Blok-Tip et al. (2005); Campbell et al. (2012)	
2 Improved clinical outcomes		
2.1 Improved psychological wellbeing and increased quality of life	• Men with ED report low self-esteem, lack of confidence and relationship issues resulting from their ED symptoms Shabsigh et al. (1998); Rosen et al. (2004); Althof et al. (2006)	• Tadalafil can increase a man’s sexual confidence via improved erectile function, and the 36-h duration of action improves spontaneity and diminishes time concerns, making it the preferred choice over sildenafil by 52%–91% of men Dean et al. (2006); Gong et al. (2017)
	• Reasons for not seeking help include embarrassment, a belief that it is a natural process of ageing or that the condition is self-limiting Gülpinar et al. (2012)	• Tadalafil can also improve the sexual and health-related quality of life of couples and normalise self-esteem Galiè et al. (2009); Seftel et al. (2009); Althof et al. (2010); Rubio-Aurioles et al. (2012); Hatzimouratidis et al. (2014); Jannini et al. (2014); Li et al. (2016)
3 Improved public health		
3.1 Increased likelihood of diagnosis of underlying health conditions	• A Danish survey of 48,910 men found 32% had ED and poor physical fitness, were smokers and obese but they did not seek medical help Paulsen et al. (2020)	• In the United Kingdom, the number of HCP consultations for CVD checks has increased since Viagra Connect has been marketed, regardless of whether men purchased it or not Lee et al. (2021)
	• This indicates that the link between ED and (CVD)/risk factors is poor in prescription-only countries despite it being an important marker Montorsi et al. (2003); Batty et al. (2010); Dong et al. (2011); Gupta et al. (2011); Averbeck et al. (2012); Tan et al. (2012)	
3.2 Increased public awareness/reduced stigmatization	• A 2020 survey of 3,032 men and women in Germany, Spain and France found 51% had poor or no understanding of ED, and 25% no knowledge of any treatments European Association of Urology (2020)	• The number of men seeking help for ED in the United Kingdom has increased since sildenafil became non-prescription Lee et al. (2021)
		• Media campaigns can play a key role in raising awareness of health conditions Wakefield et al. (2010)
4 Enhanced consumer involvement		
4.1 Empowering men to self-manage their condition	• Appropriate support and information is absent when purchasing ED treatments from unregulated websites Breau et al. (2003)	• Self-care is associated with clinical and psychological benefits and endorsed by HCPs and the European public health agenda de Silva (2011); Ludman et al. (2013)
	• A European survey found 48% of people are engaged in self-care, but 80% thought individuals should be more enabled Vintura (2021)	• Access to non-prescription medications and information in a pharmacy setting is a powerful facilitator of self-care Soller (1998); Vintura (2021)
5 Economic benefit		
5.1 Alleviation of physician consulting time and preventing later costly treatments	• There may be short-term increases in HCP resource from raised awareness of the link between ED and underlying conditions Lee et al. (2021)	• Earlier detection of diabetes and CVD would reduce longer-term health costs
		• MHRA found making sildenafil non-prescription would make more effective use of doctors’ time Medicines and Healthcare products Regulatory Agency (2017b)

**TABLE 2:** Evidence review summary of incremental risk considerations.

Risk consideration	Evidence
1 Unintended misuse	
1.1 Drug-drug interaction with nitrates	• Tadalafil plus nitrates can ↓ blood pressure (BP) Kloner et al. (2003)
	• No adverse event (AE) with single dose of 5 mg or 10 mg tadalafil H6D-LC-LVAB (2022); H6D-EW-LVCM (2022)
	• Mean max ↓4 mmHg with tadalafil 20 mg daily for 1 week H6D-EW-LVDN (2022); H6D-LC-LVBY (2022)
	• No increased risk of hypotensive cardiovascular outcome with co-possession Nunes et al. (2021)
	• Post-marketing reports identified 34 AE cases out of 84.7 million patients exposed to Cialis, seven major AE cases^a^
	• Nitrate use in Europe is declining Schwabe/Ludwig Hrsg (2021); Kloner et al. (2003); Montalescot et al. (2013); NICE (2011); Crespo-Leiro et al. (2016); NHS Digital (2015)
	• Label states not to use Cialis if taking nitrates Lilly (2021)
1.2 Drug-drug interaction with doxazosin and other alpha blockers	• Tadalafil plus alpha blockers can ↓BP
	• Use is mainly for benign prostatic hypertrophy in the United Kingdom NICE (2019), and rarely for hypertension in Germany Schwabe/Ludwig Hrsg (2021)
	• Tadalafil 20 mg + doxazosin causes ↓BP, but 5 mg does not H6D-EW-LVFG (2023); H6D-EW-LVFT (2023); H6D-EW-LVGT (2023)
	• Four double-blind studies found no difference in BP for tadalafil + alfuzosin, silodosin, or tamsulosin Goldfischer et al. (2012); Study PDY5734 (2023); H6D-EW-LVAY (2023); H6D-EW-LVGN (2023)
	• Post-marketing reports identified 225 AE cases, 17 serious^a^
	• Label states to discuss use of Cialis with pharmacist or doctor if taking alpha-blockers Lilly (2021)
1.3 Drug-drug interaction with riociguat	• Tadalafil plus riociguat can ↓BP
	• Riociguat is licensed for the treatment under specialist care of pulmonary arterial hypertension that affects 15 to 60 people per million in Europe Galiè et al. (2016)
	• Label states not to use Cialis if taking riociguat Lilly (2021)
1.4 Drug-drug interaction with antihypertensives	• Six trials found no clinically significant ↓BP for men with controlled and poorly controlled hypertension on 1–4 antihypertensives H6D-EW-LVAV (2023); H6D-EW-LVDP (2023); H6D-EW-LVBC (2023); H6D-EW-LVDS (2023); H6D-EW-LVAW (2023); H6D-EW-LVDV (2023)
	• Post-marketing reports identified 339 serious AE cases, 27 due to hypotension and 58 a major adverse cardiovascular event; most had pre-existing cardiovascular risk factors^a^
1.5 Drug-drug interaction with alcohol	• Excessive alcohol with tadalafil can cause headaches and dizziness but not lower levels of alcohol H6D-EW-LVAE (2023); H6D-EW-LVDO (2023)
1.6 Use in men with severe renal or hepatic disease	• Post-marketing data does not causally link tadalafil with a decrease in hepatic or renal function^a^
	• Daily dosing is not recommended for men with severe renal impairment because of the increased concentration of tadalafil and poor clearance H6D-EW-LVAJ (2023); H6D-EW-LVDT (2023)
2 Worsened outcomes due to self-management	
2.1 Delayed diagnosis of CVD and other underlying conditions	• 70% of men already have an underlying comorbidity diagnosed before ED Kirby et al. (2011)
	• Diabetes often remains undiagnosed for many years and PDE5 inhibitors have been shown to be beneficial, lowering all-cause mortality and incident myocardial infarction (MI) Anderson et al. (2016)
2.2 Failure to read/understand safety information in men unfit to have sex due to CVD	• Use of tadalafil is contraindicated in men with current, significant CVD Lilly (2021)
	• New adverse cardiac events are not associated with PDE5 inhibitor use for managing ED in men with significant CVD Levine et al. (2012)
3 Intentional misuse	
3.1 To enhance sexual performance	• 11% of men in a European study reported using PDE5 inhibitors without ED Corona et al. (2018)
	• 13%–27% of men who do not self-assess as having ED have mild ED Korkes et al. (2008); Harte et al. (2011)
3.2 Intentional misuse: use with ‘poppers’	• Limited data exist regarding the use of PDE5 inhibitors with concomitant illicit drugs including recreational nitrates and such use appears low Chu et al. (2003)
	•There are no post marketing concerns^a^
3.3 Use in women	•Tadalafil is not indicated for women, post marketing reports identified 267 cases^a^
4 Intentional overdose	
4.1 Use at a higher dose/frequency than on the label	• Due to its nonlinear pharmacokinetics at doses >20 mg, tadalafil exposure in case of overdose or CYP3A4 enzyme inhibition remains within safe boundaries EMTEX (2021)
5 Accidental ingestion	
5.1 Accidental ingestion by children	• Post-marketing data report 14 cases, 2 serious with full recovery^a^

^a^ Post-marketing adverse event reports from 84,674,000 patients exposed to Cialis worldwide from 2002 to 2020 (EMTEX, 2021).

## 3 Results

### 3.1 Incremental benefit ratings

Each incremental benefit was scored in two rounds of ratings, Table 3. Shaded boxes indicate where consensus was reached (all scores within 1 point of each other). According to the second round of scoring, the expert panel considered that all incremental benefits were likely, with the exception of one expert who did not think there would be an increased likelihood of diagnosis of underlying health conditions. Individual scores ranged from low to high with mean likelihood scores of 1.7–2.3 but there was no consensus.

**TABLE 3:** Incremental benefit scores.

Incremental benefit	Round	Likelihood					Clinical impact					Mean likelihood	Mean clinical impact	Overall attribute
		0	1	2	3	D	0	1	2	3	D	(0–3)	(0–3)	(0–9)
1 Improved access														
1.1 Decrease in barriers to treatment	1st	1		2	3		1		3	2		2.2	2.0	4.3
	2nd		1	2	3			1	2	3		2.3	2.3	5.4
1.2 Less use of counterfeit medicines	1st	1		4	1				3	3		1.8	2.5	4.6
	2nd		1	4	1				3	3		2.0	2.5	5.0
2 Improved clinical outcomes														
2.1 Improved psychological wellbeing and increased quality of life	1st		3	1	2			1	3	2		1.8	2.2	4.0
	2nd		2	1	3			2	3	1		2.2	1.8	4.0
3 Improved public health														
3.1 Increased likelihood of diagnosis of underlying health conditions	1st	1	2	1	2			2	3	1		1.7	1.8	3.1
	2nd	1	2	1	2			3	2	1		1.7	1.7	2.8
3.2 Increased public awareness/reduced stigmatisation	1st		3		3			2	3	1		2.0	1.8	3.7
	2nd		2	1	3			2	3	1		2.2	1.8	4.0
4 Enhanced consumer involvement														
4.1 Empowering men to self-manage their condition	1st		1	4	1			1	5			2.9	1.8	3.7
	2nd		1	4	1			1	5			2.0	1.8	3.7
5 Economic benefit														
5.1 Alleviation of physician consulting time and preventing later costly treatments	1st		2	2	2			3	1	2		2.0	1.8	3.7
	2nd		3	2	1			2	4			1.7	1.7	2.8

D = do not know; Shaded boxes = consensus reached.

Likelihood mean score: 0, the behaviour will almost never occur; 3, the behaviour is likely to be frequent.

Clinical impact mean score: 0, there would be no clinical impact; 3, the clinical impact would be high.

There was consensus of a moderate to high clinical impact if there were less use of counterfeit medicines (2.5). There was also agreement of a moderate clinical impact from empowering men to self-manage their condition (1.8) and for the economic benefits (1.7). For the other incremental benefits, consensus was not reached but the mean clinical impact ranged from 1.7 to 2.3.

Overall attribute scores ranged from 2.8 to 5.4, with the highest scores for a decrease in barriers to treatment (5.4), less use of counterfeit medicines (5.0), improved psychological wellbeing and increased quality of life (4.0), and increased public awareness and reduced stigmatisation (4.0).

### 3.2 Incremental risk ratings

There was consensus amongst experts that the risks were low/unlikely to occur and would not have a significant clinical impact. The information on the outer carton was considered to sufficiently manage the risks.

Consensus was reached on how likely 12 out of 14 incremental risks were to occur and of a low clinical impact for 11 of them (Table 4, shaded boxes). The overall attribute scores were much lower than for the benefits, ranging from 0.2 to 2.2, with 11 risks scoring 1.0 or less.

**TABLE 4:** Incremental risk scores.

Incremental risk	Round	Likelihood					Clinical impact					Mean likelihood	Mean clinical impact	Overall attribute
		0	1	2	3	D	0	1	2	3	D	(0–3)	(0–3)	(0–9)
1 Unintended misuse														
1.1 Drug-drug interaction with nitrates	1st	1	5					2	4			0.8	1.7	1.4
1.2A Drug-drug interaction with doxazosin	1st	3	3				2	4				0.5	0.7	0.3
1.2B Drug-drug interaction with other alpha blockers	1st	2	4				2	4				0.7	0.7	0.4
1.3 Drug-drug interaction with riociguat	1st	1	4	1			1	4	1			1.0	1.0	1.0
	2nd		4	2				2	4			1.3	1.7	2.2
1.4 Drug-drug interaction with antihypertensives	1st	2	4				1	4	1			0.7	1.0	0.7
	2nd	2	4				2	4				0.7	0.7	0.4
1.5 Drug-drug interaction with alcohol	1st	1	4	1			2	4				1.0	0.7	0.7
	2nd	1	5				2	4				0.8	0.7	0.6
1.6 Use in men with severe renal or hepatic disease	1st	1	5				1	5				0.8	0.8	0.7
2 Worsened outcomes due to self-management														
2.1 Delayed diagnosis of CVD and other underlying conditions	1st	2	3		1		3	2	1			1.0	0.7	0.7
	2nd	1	4	1			2	3	1			1.0	0.8	0.8
2.2 Failure to read/understand safety information in men unfit to have sex due to CVD	1st		4	1	1			6				1.5	1.0	1.5
	2nd		5			1		5			1	1.0	1.0	1.0
3 Intentional misuse														
3.1 To enhance sexual performance	1st		1	3	2		1	5				2.2	0.8	1.8
	2nd			4	2		2	4				2.3	0.7	1.6
3.2 Intentional misuse: use with “poppers”	1st	1	4		1		1	5				1.2	0.8	1.0
	2nd		5	1			1	5				1.2	0.8	1.0
3.3 Use in women	1st	2	4				4	2				0.7	0.3	0.2
4 Intentional overdose														
4.1 Use at a higher dose/frequency than on the label	1st		2	3	1		1	4		1		1.8	1.2	2.1
	2nd		1	4	1		3	3				2.0	0.5	1.0
5 Accidental ingestion														
5.1 Accidental ingestion by children	1st	1	5				2	2	2			0.8	1.0	0.8
	2nd	2	4				2	4				0.7	0.7	0.4

D = do not know; Shaded boxes = consensus reached.

Likelihood mean score: 0, the behaviour will almost never occur; 3, the behaviour is likely to be frequent.

Clinical impact mean score: 0, there would be no clinical impact; 3, the clinical impact would be high.

An increase in drug-drug interactions were deemed unlikely for tadalafil with nitrates due to their low and decreasing usage in Germany (0.8); in 2020, there were 53 million defined daily doses (DDD) of long-acting nitrates prescribed (reduced from 233 million DDDs in 2011), 52 million DDDs of molsidomine and 31 million DDDs of glyceryl trinitrate. (Schwabe/Ludwig Hrsg, 2021). In the context of this reducing use, the warning on the package (see below) was considered sufficient mitigation for this risk.

**IMPORTANT**: Do not use if you are taking either of the following two medicines:

• *Nitrate medicine for chest pain (angina pectoris) or heart failure*
• *Riociguat for high blood pressure in the lungs*

The warning messages in the PIL are:

What you need to know before you take *TM* (*TM* is a placeholder for the approved brand name).

Do not take *TM* if you:

Take any medicines called nitrates or nitric oxide donors (such as glyceryl trinitrate, isosorbide mononitrate, isosorbide dinitrate for the relief of chest pain or heart failure, or amyl nitrite also known as “poppers”, nicorandil, molsidomine or sodium nitroprusside).

These are often used for the relief of chest pain (angina pectoris), or heart failure.

Using *TM* with any of these medicines may lead to a dangerous fall in blood pressure.

If it were to occur, the mean clinical impact score was rated low to moderate (1.7). All experts agreed there was very low likelihood of an increase in drug-drug interaction with doxazosin (0.5) or other alpha-blockers (0.7) and that there would be a minimal clinical impact (0.7) as they are not regularly used in Germany for hypertension. In 2020, there were 123 million defined daily doses (DDDs) of alpha blockers used to treat hypertension compared with 2,589 million DDDs of calcium channel blockers and 6,050 million DDDs of ACE inhibitors (Schwabe/Ludwig Hrsg, 2021). Similarly, there was low concern about any increase in interaction with other antihypertensives, with an overall attribute score of 0.4 because of the evidence of extremely few cases of such complications in the post-marketing safety data for 84,674,000 patients who have been exposed to Cialis worldwide from 2002 to 15 October 2020. (EMTEX, 2021).

The expert panel viewed the likelihood of an increase in interaction with riociguat a little higher because of the recent approval for treatment of post thrombotic pulmonary hypertension meaning more men could be at risk. The mean clinical impact score of 1.7 and overall attribute score of 2.2 reflected a moderate level of concern, but being under the care of a specialist and the clear warning messages on the pack (shown above) and in the PIL (see below) were felt to be strong mitigating factors. The warning message in the PIL is:

Do not take *TM* if you are taking a medicine called riociguat (or other medicines of a group called guanylate cyclase stimulators), used to treat pulmonary arterial hypertension (i.e., high blood pressure in the lungs) and chronic thromboembolic pulmonary hypertension (i.e., high blood pressure in the lungs due to blood clots).

There was agreement that the likelihood of alcohol interaction occurring more frequently if tadalafil were available without prescription was low, as was the clinical impact with an overall score of 0.6, reflecting the evidence from clinical pharmacology studies that was reviewed by the experts during the meeting.

The experts also agreed that men may be more likely to use tadalafil to enhance sexual performance (2.3), but that this would have a low clinical impact (0.7) as there was no evidence to suggest that the risks to health from use to enhance clinical performance would be clinically significant. There were mixed opinions on whether intentional use at a higher dose/frequency would increase, but consensus was that this would have a low clinical impact (0.5) according to the evidence and patient reported experiences.

The overall attribute scores reflected consensus of a low likelihood or clinical impact of increased use in men with severe renal or hepatic disease (0.7), failure to read/understand safety information for men unfit to have sex due to cardiovascular disease (CVD) (1.0), use with “poppers” (1.0), use in women (0.2) or accidental ingestion by children (0.4). All considerations of the low clinical impact were based on evidence from published studies and 18 years of post-marketing safety data for patients exposed to Cialis worldwide that were reviewed by the experts in the meeting.

Finally, there were differing views on the likelihood (1.0) and clinical impact (0.8) of delayed diagnosis of CVD and other underlying conditions. If ED were the only early symptom of CVD, it was felt to be unlikely that a man would arrange to see a cardiologist, therefore taking tadalafil would not delay a diagnosis of CVD. However, self-treating ED may reduce the likelihood of seeing an urologist and so could lead to a delay in diagnosis of an underlying urological condition. On balance, the overall attribute of this scenario was scored low (0.7).

### 3.3 Risk mitigation measures

If tadalafil were to be reclassified to non-prescription status in Germany, training would be provided to pharmacists highlighting the contraindications, potential drug-drug interactions and conditions where men should first consult their physician. Messages on the carton and patient information leaflet (PIL) would include contraindications, safety information, dosage and side effects. These messages have been tested in men in three separate studies to ensure comprehension. The expert panel felt that these measures were appropriate and sufficient to mitigate against the identified incremental risks and that the results of the studies demonstrated that the information was sufficiently well understood and that consumers would make appropriate self-selection decisions. An additional suggested measure was to add a warning regarding the potential drug-drug interaction with veriziguat which was recently approved for chronic heart failure.

## 4 Discussion

The Brass decision analysis tool worked well in terms of achieving the objectives of the meeting. The identification of the incremental benefits and risks and collation of evidence from a diverse range of information sources including clinical trials, real-world studies and patient safety data was helpful in providing an evidence-based foundation to facilitate a comprehensive and rational assessment of the benefit-risk analysis. The Group Delphi technique provided the opportunity for divergent opinions to be frankly discussed and the experiences and expertise of the different specialists to be presented. In many cases, this led to narrowing of the range of scores where a second round of voting occurred, thereby gaining or moving towards consensus.

The lack of consensus regarding the degree of likelihood and clinical impact of the majority of identified incremental benefits may in part be due to the complex number of factors involved in achieving each benefit. Importantly, the discussion about benefits is hypothetical as there is no PDE5 inhibitor available without prescription in Germany. Data from the UK regarding the experience with Viagra Connect was presented, but this was not felt to be directly applicable because of the differences in healthcare systems. For safety, patient safety update reports and large observational studies were able to provide strong evidence to help quantify the risks and are likely to have contributed to the high level of consensus reached.

It is possible that gaps in evidence or differing opinions on whether evidence from different healthcare systems would be replicable in Germany were factors in the range of scores for the benefits. For instance, some of the evidence for a reduction in barriers to treatment and improved diagnosis of underlying conditions came from the United Kingdom. Germany has substantially more urologists and therefore easier patient access to them. There were also differences of opinion regarding the level of health consultations that take place in pharmacies in Germany compared to the United Kingdom. However, there was general agreement that people are more likely to access services when provided locally, such as the increased take-up of flu vaccinations when provided at pharmacies.

From the patient’s perspective it is more convenient to go straight to the pharmacy to get the medication rather than having to go to the doctor first for a prescription. There was some agreement that men may not want to see a doctor for ED because of embarrassment and therefore prefer to buy PDE5 inhibitors from a pharmacy. Recent TV advertising was believed to have raised awareness of ED and reduced inhibition thresholds to seek treatment. If tadalafil were prescription-free, it was considered likely that the topic of ED would be more publicly discussed, and the feelings of embarrassment reduced.

It was widely agreed that the consumer would be unaware if the product was counterfeited or not, but if it were available to buy in pharmacies, patients would have less reason to go to unreliable internet sources. The New Zealand example of reduced counterfeit packages following reclassification was discussed and considered to be potentially applicable in Germany.

Only three risks scored above 1, the potential drug-drug interaction with riociguat which scored 2.2, potential misuse by men without ED which scored 1.6 and use with nitrates which scored 1.3. Riociguat is a relatively new drug used in the treatment of the rare condition of pulmonary arterial hypertension or more recently chronic thromboembolic pulmonary hypertension. The score reflected the moderate degree of clinical impact such a drug-drug reaction would have, but there was general agreement that this scenario would be unlikely because men would be under specialist clinical supervision and would also see the risk mitigation warning messages on the packaging and PIL.

Potential misuse by men without ED was considered moderately likely to increase if tadalafil were to be available without prescription, but the clinical impact of this overuse was deemed to be low.

The risk of drug-drug interactions with nitrates was considered of low likelihood and to have low clinical impact, largely because they are now rarely used in Germany. The warnings on the package and label were also felt to be sufficient though the panel recommended that men be directed to discuss their medication with a pharmacist or doctor if they are unsure about what they are taking.

Across the domains there were no additional risk mitigation measures considered to be essential beyond the wording on the carton and in the PIL. There were suggestions to include warnings about newer medications for heart failure, such as vericiguat on the packaging and PIL. The discussion highlighted points about drug-drug interactions in general and the balance required in terms of how much information to include on the PIL, such as the trade and generic names. It was agreed that discussion with a GP or pharmacist about any potential interactions with their other medication would be beneficial and that this could be a recommendation in the PIL.

## 5 Conclusion

Based on this thorough and systematic assessment of the evidence for the incremental benefits and risks of making tadalafil available without prescription in Germany, the consensus amongst clinical experts was that the incremental risks would be small. They would either be unlikely to occur or, if more likely to occur, would have little clinical impact. There was high level agreement that the risks are sufficiently manageable, so even if there were differing opinions regarding the magnitude of benefits, the average risk evaluation appeared significantly lower than the potential benefits.
